# Wrapped: An R package for circular data

**DOI:** 10.1371/journal.pone.0188512

**Published:** 2017-12-07

**Authors:** Saralees Nadarajah, Yuanyuan Zhang

**Affiliations:** School of Mathematics, University of Manchester, Manchester, M13 9PL, United Kingdom; Janssen Research and Development, UNITED STATES

## Abstract

The Wrapped package computes the probability density function, cumulative distribution function, quantile function and also generates random samples for many univariate wrapped distributions. It also computes maximum likelihood estimates, standard errors, confidence intervals and measures of goodness of fit for nearly fifty univariate wrapped distributions. Numerical illustrations of the package are given.

## Introduction

Circular data are data recorded in degrees or radii. They arise in a wide variety of scientific areas. Some published applications involving real circular data include: data from fibre composites and from ceramic foams [1]; skeletal representations in medical image analysis and biomechanical gait analysis of the knee joint [2]; worldwide earthquakes with magnitude greater than or equal to 7.0 M W [3]; wave direction data in the Adriatic sea, off the coast of Italy [4]; data set of phase angles of circadian-related genes in heart and liver tissues [5].

Wrapping is a popular method for constructing distributions for circular data. Let *g* denote a valid probability density function (PDF) defined on the real line. Let *G* denote the corresponding cumulative distribution function (CDF). The wrapped distribution corresponding to *g* and *G* has the PDF and CDF specified by

$$\begin{array}{c} f\left(x\right)=\sum_{k=-\infty }^{\infty }g(x+2\pi k) \end{array}$$ (1)

and

$$\begin{array}{c} F\left(x\right)=\sum_{k=-\infty }^{\infty }[G(x+2\pi k)-G(-\pi +2\pi k)], \end{array}$$ (2)

respectively. *g* could also be chosen as a PDF on the positive real line or as a probability mass function of a discrete random variable. But usually circular data are recorded between −*π* and *π*. So, we stick to (1) and (2).

Many wrapped distributions have been proposed and studied in the literature. These include the wrapped normal distribution [6], wrapped Cauchy distribution [7], wrapped skew normal distribution [8], wrapped exponential and Laplace distributions [9], wrapped stable distribution [10], wrapped gamma distribution [11], wrapped *t* distribution [12], wrapped lognormal and Weibull distributions [13], wrapped skew Laplace distribution [14], wrapped weighted exponential distribution [15], wrapped hypo exponential distribution [16], wrapped geometric distribution [17], wrapped Poisson distribution [18], wrapped zero inflated Poisson distribution [19] and wrapped Lindley distribution [20]. There are also a number of R [21] packages developed to implement wrapped distributions including: NPCirc [22] giving procedures for wrapped Cauchy, wrapped normal and wrapped skew normal distributions; wle [23] giving procedures for the wrapped normal distribution; circular [24] giving procedures for wrapped normal, wrapped Cauchy and wrapped stable distributions; CircStats [25] giving procedures for wrapped normal, wrapped Cauchy and wrapped stable distributions; BAMBI [26] giving procedures for wrapped normal and wrapped normal mixtures distributions; movehMM [27] giving procedures for the wrapped Cauchy distribution; SpaDES [28] giving procedures for the wrapped normal distribution.

But we are not aware of an R package applicable for computing (1) and (2) for given parametric forms for *g* and *G*. The aim of this paper is to introduce an R package developed by the authors that is applicable for computing many wrapped distributions. The package is named Wrapped [29]. The package performs the following:

i) computes (1), (2) and the corresponding quantile function for given parametric forms for *g* and *G*. Because (1) and (2) are infinite sums, the following approximations are used
  $$\begin{array}{c} f\left(x\right)\approx \sum_{k=-K}^{K}g(x+2\pi k) \end{array}$$ (3)
  and
  $$\begin{array}{c} F\left(x\right)\approx \sum_{k=-K}^{K}[G(x+2\pi k)-G(-\pi +2\pi k)], \end{array}$$ (4)
  where *K* ≥ 1 is an integer. We shall refer to (3) and (4) as the approximate PDF and approximate CDF, respectively.
ii) generates random samples from the wrapped distribution for given parametric forms for *g* and *G*.
iii) computes maximum likelihood estimates of the parameters, standard errors, 95 percent confidence intervals, value of Cramer von Mises statistic, value of Anderson Darling statistic, value of Kolmogorov Smirnov test statistic and its *p*-value, value of Akaike Information Criterion, value of Consistent Akaike Information Criterion, value of Bayesian Information Criterion, value of Hannan Quinn Information Criterion, minimum value of the negative log likelihood function and convergence status when some data are fitted by one of the following wrapped distributions: wrapped normal, wrapped Gumbel, wrapped logistic, wrapped Student’s *t*, wrapped Cauchy, wrapped skew normal, wrapped skew *t*, wrapped skew Cauchy, wrapped asymmetric Laplace, wrapped normal Laplace, wrapped generalized logistic, wrapped skew Laplace, wrapped exponential power, wrapped skew exponential type 1, wrapped skew exponential type 2, wrapped skew exponential type 3, wrapped skew exponential type 4, wrapped normal exponential *t*, wrapped skew normal type 2, wrapped ex Gaussian, wrapped skew *t* type 1, wrapped skew *t* type 3, wrapped skew *t* type 4, wrapped skew *t* type 5, wrapped sinh arcsinh, wrapped exponential generalized beta type 2, wrapped Johnson’s *S*_*u*_, wrapped skew generalized *t*, wrapped skew hyperbolic, wrapped asymmetric Laplace, wrapped polynomial tail Laplace, wrapped generalized asymmetric *t*, wrapped variance gamma, wrapped normal inverse Gaussian, wrapped hyperbolic, wrapped skew Laplace, wrapped slash, wrapped beta normal, wrapped Laplace, wrapped short tailed symmetric and wrapped log gamma distributions.
iv) plots PDFs, CDFs, quantile functions and histograms of the radii of 100 random numbers for a specified wrapped distribution. The wrapped distribution must be one of those mentioned in iii).

A description of the program structure of the package is given in the next section. Some numerical illustrations of the package are given in the following section. The paper concludes with a discussion section.

## Program structure

The following are the command syntaxes of the Wrapped package:

dwrappedg(x, spec, K = K,…)

pwrappedg(x, spec, K = K,…)

rwrappedg(n, spec,…)

qwrappedg(p, spec, K = K,…)

mwrappedg(g, data, starts, K = K, method = “BFGS”)

plotfour(g, K = K, para, plotit)

dwrappedg computes the approximate PDF for given x, g and K. The g must be specified through the string spec. For example, if spec = “norm” then g is the standard normal PDF. If parameters are needed to specify spec they can be supplied through … For example, spec = “norm” with … replaced by mean = 1, sd = 10 will mean that g is a normal PDF with unit mean and standard deviation 10. pwrappedg computes the approximate CDF for given x, g and K. For both dwrappedg and pwrappedg, x can be a scalar or a vector.

qwrappedg computes the roots of

$$\begin{array}{c} \sum_{k=-K}^{K}[G(x+2\pi k)-G(-\pi +2\pi k)]=p \end{array}$$

for given p, g and K. p can be a scalar or a vector.

rwrappedg generates n random numbers from the wrapped distribution specified by g. The random numbers are cos(rspec(n,…)), cos(rspec(n,…)).

To use dwrappedg, pwrappedg and rwrappedg, one must have the functions dg, pg, qg and rg available in the base package of R or one of its contributed packages. In the latter case, the relevant contributed package(s) must be first downloaded. For example, one must have the functions dnorm, pnorm, qnorm and rnorm available for computing the wrapped normal distribution. These functions are indeed available in the base package of R.

mwrappedg fits the wrapped distribution specified by g and K to the data contained in data. starts is a vector of starting values for the parameters of g in the stated order. For example, if g = “norm” then starts = c(0, 1) gives the starting values of 0 for the mean and 1 for the standard deviation of the wrapped normal distribution. The following choices are possible for g:

- Normal distribution [30, 31]: g = “norm”, starts = c(a, b) and
  $$\begin{array}{c} g\left(x\right)=\frac{1}{\sqrt{2\pi }b}\exp[-\frac{{\left(x-a\right)}^{2}}{2b^{2}}] \end{array}$$
  for −∞ < *x* < +∞, −∞ < *a* < +∞ and *b* > 0.
- Gumbel distribution [32]: g = “gumbel”, starts = c(a, b) and
  $$\begin{array}{c} g\left(x\right)=\frac{1}{b}\exp(-\frac{x-a}{b})\exp[-\exp(-\frac{x-a}{b})] \end{array}$$
  for −∞ < *x* < +∞, −∞ < *a* < +∞ and *b* > 0. The contributed R package evd due to [33] is used to compute this PDF *g*.
- Logistic distribution: g = “logis”, starts = c(a, b) and
  $$\begin{array}{c} g\left(x\right)=\frac{1}{b}\text{exp}\left(-\frac{x-a}{b}\right){\left[1+\text{exp}\left(-\frac{x-a}{b}\right)\right]}^{-2} \end{array}$$
  for −∞ < *x* < +∞, −∞ < *a* < +∞ and *b* > 0.
- Student’s *t* distribution [34]: g = “t.scaled”, starts = c(a, b, n) and
  $$\begin{array}{c} g\left(x\right)=\frac{\Gamma \left(\frac{n+1}{2}\right)}{b\sqrt{n\pi }\Gamma \left(\frac{n}{2}\right)}{\left[1+\frac{{\left(x-a\right)}^{2}}{nb^{2}}\right]}^{-\frac{n+1}{2}} \end{array}$$
  for −∞ < *x* < +∞, −∞ < *a* < +∞, *b* > 0 and *n* > 0, where Γ(⋅) denotes the gamma function defined by
  $$\begin{array}{c} \Gamma \left(a\right)=\int_{0}^{+\infty }t^{a-1}\exp\left(-t\right)dt. \end{array}$$
  The contributed R package metRology due to [35] is used to compute this PDF *g*.
- Cauchy distribution [34]: g = “cauchy”, starts = c(a, b) and
  $$\begin{array}{c} g\left(x\right)=\frac{1}{b\pi }{\left[1+\frac{{\left(x-a\right)}^{2}}{b^{2}}\right]}^{-1} \end{array}$$
  for −∞ < *x* < +∞, −∞ < *a* < +∞ and *b* > 0.
- Skew normal distribution [36]: g = “sn”, starts = c(a, b, c) and
  $$\begin{array}{c} g\left(x\right)=2\phi \left(\frac{x-a}{b}\right)\Phi \left(c\frac{x-a}{b}\right) \end{array}$$
  for −∞ < *x* < +∞, −∞ < *a* < +∞, *b* > 0 and −∞ < *c* < +∞, where *ϕ*(⋅) and *Φ*(⋅) denote, respectively, the PDF and CDF of the standard normal distribution defined by
  $$\begin{array}{c} \phi \left(x\right)=\frac{1}{\sqrt{2\pi }}\exp(-\frac{x^{2}}{2}) \end{array}$$
  and
  $$\begin{array}{c} \Phi \left(x\right)=\int_{-\infty }^{x}\phi \left(y\right)dy, \end{array}$$
  respectively. The contributed R package sn due to [37] is used to compute this PDF *g*.
- Skew *t* distribution of type 2 [38]: g = “st”, starts = c(a, b, c, n) and
  $$\begin{array}{c} g\left(x\right)=\frac{2\Gamma \left(\frac{n+2}{2}\right)}{b\pi \sqrt{n\left(n+1\right)}\Gamma \left(\frac{n}{2}\right)}{\left[1+\frac{{\left(x-a\right)}^{2}}{nb^{2}}\right]}^{-\frac{n+1}{2}}{\int_{-\infty }^{x^{*}}\left[1+\frac{y^{2}}{n+1}\right]}^{-\frac{n+2}{2}}dy \end{array}$$
  for −∞ < *x* < +∞, −∞ < *a* < +∞, *b* > 0, −∞ < *c* < +∞ and *n* > 0, where $x^{*}=c\frac{x-a}{b}\sqrt{\frac{n+1}{{\left(x-a\right)}^{2}+n}}$. The contributed R package sn due to [37] is used to compute this PDF *g*.
- Skew Cauchy distribution [38]: g = “sc”, starts = c(a, b, c, n) and
  $$\begin{array}{c} g\left(x\right)=\frac{1}{b\pi \sqrt{2}}{\left[1+\frac{{\left(x-a\right)}^{2}}{nb^{2}}\right]}^{-1}{\int_{-\infty }^{x^{*}}\left[1+\frac{y^{2}}{2}\right]}^{-\frac{3}{2}}dy \end{array}$$
  for −∞ < *x* < +∞, −∞ < *a* < +∞, *b* > 0, −∞ < *c* < +∞ and *n* > 0, where $x^{*}=c\frac{x-a}{b}\sqrt{\frac{2}{{\left(x-a\right)}^{2}+1}}$. The contributed R package sn due to [37] is used to compute this PDF *g*.
- Asymmetric Laplace distribution [39]: g = “ALD”, starts = c(a, b, p) and
  $$\begin{array}{c} g\left(x\right)=\frac{p(1-p)}{b}\exp[-\rho_{p}(\frac{x-a}{b})] \end{array}$$
  for −∞ < *x* < +∞, −∞ < *a* < +∞, *b* > 0 and 0 < *p* < 1, where *ρ*_*p*_(*u*) = *u*[*p* − *I*{*u* < 0}] and *I* {⋅} denotes the indicator function defined by *I* {*A*} = 1 if *A* is true and *I* {*A*} = 0 if *A* is false. The contributed R package ald due to [40] is used to compute this PDF *g*.
- Normal Laplace distribution [41]: g = “nl”, starts = c(a, b, c, d) and
  $$\begin{array}{c} g\left(x\right)=\frac{cd}{c+d}\phi (\frac{x-a}{b})[R(cb-\frac{x-a}{b})+R(db+\frac{x-a}{b})] \end{array}$$
  for −∞ < *x* < +∞, −∞ < *a* < +∞, *b* > 0, *c* > 0 and *d* > 0, where *R*(*u*) = [1 − *Φ*(*u*)]/*ϕ*(*u*). The contributed R package ald due to [42] is used to compute this PDF *g*.
- Generalized logistic distribution [43]: g = “glogis”, starts = c(a, b, c) and
  $$\begin{array}{c} g\left(x\right)=\frac{c}{b}\text{exp}\left(-\frac{x-a}{b}\right){\left[1+\text{exp}\left(-\frac{x-a}{b}\right)\right]}^{-c-1} \end{array}$$
  for −∞ < *x* < +∞, −∞ < *a* < +∞, *b* > 0, and *c* > 0. The contributed R package glogis due to [44] is used to compute this PDF *g*.
- Skew Laplace distribution [45]: g = “sld”, starts = c(a, b, c) and
  $$\begin{array}{c} G^{-1}\left(p\right)=a+b[(1-c)\log p-c\log(1-p)] \end{array}$$
  for 0 < *p* < 1, −∞ < *a* < +∞, *b* > 0, and 0 < *c* < 1. The contributed R package sld due to [46] is used to compute this PDF *g*.
- Exponential power distribution: g = “normp”, starts = c(a, b, c) and
  $$\begin{array}{c} g\left(x\right)=\frac{1}{2bc^{1/c}\Gamma \left(1+\frac{1}{c}\right)}\text{exp}\left[-\frac{1}{c}{\left(\frac{x-a}{b}\right)}^{c}\right] \end{array}$$
  for −∞ < *x* < +∞, −∞ < *a* < +∞, *b* > 0 and *c* > 0. The contributed R package normalp due to [47] is used to compute this PDF *g*.
- Skew exponential type 1 distribution [48]: g = “SEP1”, starts = c(a, b, c, d) and
  $$\begin{array}{c} g\left(x\right)=\frac{1}{2bd^{\frac{2}{d}-2}\Gamma^{2}\left(\frac{1}{d}\right)}\text{exp}\left[-\frac{1}{d}{\left|\frac{x-a}{b}\right|}^{d}]\int_{-\infty }^{c\left(x-a\right)/b}\text{exp}[-\frac{1}{d}{\left|y\right|}^{d}\right]dy \end{array}$$
  for −∞ < *x* < +∞, −∞ < *a* < +∞, *b* > 0, −∞ < *c* < +∞ and *d* > 0. The contributed R package gamlss.dist due to [49] is used to compute this PDF *g*.
- Skew exponential type 2 distribution [48]: g = “SEP2”, starts = c(a, b, c, d) and
  $$\begin{array}{c} g\left(x\right)=\frac{1}{bd^{\frac{1}{d}-1}\Gamma \left(\frac{1}{d}\right)}\text{exp}\left[-\frac{1}{d}{\left|\frac{x-a}{b}\right|}^{d}\right]\Phi \left(c\sqrt{\frac{2}{d}}\;\text{sign}\left(\frac{x-a}{b}\right){\left|\frac{x-a}{b}\right|}^{d/2}\right) \end{array}$$
  for −∞ < *x* < +∞, −∞ < *a* < +∞, *b* > 0, −∞ < *c* < +∞ and *d* > 0. The contributed R package gamlss.dist due to [49] is used to compute this PDF *g*.
- Skew exponential type 3 distribution [48]: g = “SEP3”, starts = c(a, b, c, d) and
  $$\begin{array}{ccc} g\left(x\right) & = & \frac{cd}{b2^{\frac{1}{d}}\left(1+c^{2}\right)\Gamma \left(\frac{1}{d}\right)}\{\text{exp}\left[-\frac{1}{2}{\left|c\frac{x-a}{b}\right|}^{d}\right]I\left\{x<a\right\}\\ & & +\text{exp}\left[-\frac{1}{2}{\left|\frac{x-a}{cb}\right|}^{d}\right]I\left\{x\geq a\right\}\} \end{array}$$
  for −∞ < *x* < +∞, −∞ < *a* < +∞, *b* > 0, *c* > 0 and *d* > 0. The contributed R package gamlss.dist due to [49] is used to compute this PDF *g*.
- Skew exponential type 4 distribution [48]: g = “SEP4”, starts = c(a, b, c, d) and
  $$\begin{array}{ccc} g\left(x\right) & = & \frac{1}{b\left[\Gamma \left(1+\frac{1}{c}\right)+\Gamma \left(1+\frac{1}{d}\right)\right]}\{\text{exp}\left[-{\left|\frac{x-a}{b}\right|}^{c}\right]I\left\{x<a\right\}\\ & & +\text{exp}\left[-{\left|\frac{x-a}{b}\right|}^{d}\right]I\left\{x\geq a\right\}\} \end{array}$$
  for −∞ < *x* < +∞, −∞ < *a* < +∞, *b* > 0, *c* > 0 and *d* > 0. The contributed R package gamlss.dist due to [49] is used to compute this PDF *g*.
- Normal exponential *t* distribution [48]: g = “NET”, starts = c(a, b, c, d) and
  $$\begin{array}{c} g\left(x\right)=\{\begin{array}{cc} \exp[-\frac{{\left(x-a\right)}^{2}}{2b^{2}}], & \text{if}\;|\frac{x-a}{b}|\leq c\text{,}\\ \exp[-c|\frac{x-a}{b}|+\frac{c^{2}}{2}], & \text{if}\;c<|\frac{x-a}{b}|\leq d\text{,}\\ \exp[-cd\log|\frac{x-a}{bd}|-cd+\frac{c^{2}}{2}], & \text{if}\;|\frac{x-a}{b}|>d \end{array} \end{array}$$
  for −∞ < *x* < +∞, −∞ < *a* < +∞, *b* > 0, *c* > 1 and *d* > *c*. The contributed R package gamlss.dist due to [49] is used to compute this PDF *g*.
- Skew normal type 2 distribution [48]: g = “SN2”, starts = c(a, b, c) and
  $$\begin{array}{c} g\left(x\right)=\frac{2c}{\sqrt{2\pi }b\left(1+c^{2}\right)}\{\text{exp}\left[-\frac{c^{2}{\left(x-a\right)}^{2}}{2b^{2}}\right]I\left\{x<a\right\}+\text{exp}\left[-\frac{{\left(x-a\right)}^{2}}{2b^{2}c^{2}}\right]I\left\{x\geq a\right\}\} \end{array}$$
  for −∞ < *x* < +∞, −∞ < *a* < +∞, *b* > 0 and *c* > 0. The contributed R package gamlss.dist due to [49] is used to compute this PDF *g*.
- Ex Gaussian distribution [48]: g = “exGAUS”, starts = c(a, b, c) and
  $$\begin{array}{c} g\left(x\right)=\frac{1}{c}\exp(-\frac{a-x}{c}+\frac{b^{2}}{2c^{2}})\Phi \exp(\frac{x-a}{b}-\frac{b}{c}) \end{array}$$
  for −∞ < *x* < +∞, −∞ < *a* < +∞, *b* > 0 and *c* > 0. The contributed R package gamlss.dist due to [49] is used to compute this PDF *g*.
- Skew *t* distribution of type 1 [50]: g = “ST1”, starts = c(a, b, n, c) and
  $$\begin{array}{c} g\left(x\right)=\frac{2\Gamma^{2}\left(\frac{n+1}{2}\right)}{b\pi^{2}n\Gamma^{2}\left(\frac{n}{2}\right)}{\left[1+\frac{{\left(x-a\right)}^{2}}{nb^{2}}\right]}^{-\frac{n+1}{2}}{\int_{-\infty }^{x^{*}}\left[1+\frac{y^{2}}{n}\right]}^{-\frac{n+1}{2}}dy \end{array}$$
  for −∞ < *x* < +∞, −∞ < *a* < +∞, *b* > 0, −∞ < *c* < +∞ and *n* > 0, where $x^{*}=c\frac{x-a}{b}\sqrt{\frac{n+1}{{\left(x-a\right)}^{2}+n}}$. The contributed R package gamlss.dist due to [49] is used to compute this PDF *g*.
- Skew *t* distribution of type 3 [51]: g = “ST3”, starts = c(a, b, c, d) and
  $$\begin{array}{c} g\left(x\right)=\frac{2c}{b^{2}\sqrt{d}\left(1+c^{2}\right)B\left(\frac{1}{2},\frac{d}{2}\right)}\{1+\frac{{\left(x-a\right)}^{2}}{b^{2}d}\left[c^{2}I\left\{x<a\right\}+\frac{1}{c^{2}}I\left\{x\geq a\right\}\right]\} \end{array}$$
  for −∞ < *x* < +∞, −∞ < *a* < +∞, *b* > 0, *c* > 0 and *d* > 0, where *B*(⋅, ⋅) denotes the beta function defined by
  $$\begin{array}{c} B\left(a,b\right)=\int_{0}^{1}t^{a-1}{\left(1-t\right)}^{b-1}dt. \end{array}$$
  The contributed R package gamlss.dist due to [49] is used to compute this PDF *g*.
- Skew *t* distribution of type 4: g = “ST4”, starts = c(a, b, c, d) and
  $$\begin{array}{c} g\left(x\right)=\frac{1}{\sqrt{c}B\left(\frac{1}{2},\frac{c}{2}\right)+\sqrt{d}B\left(\frac{1}{2},\frac{d}{2}\right)}\{{\left[1+\frac{{\left(x-a\right)}^{2}}{b^{2}c}\right]}^{-\frac{1+c}{2}}I\left\{x<a\right\}\\ +{\left[1+\frac{{\left(x-a\right)}^{2}}{b^{2}d}\right]}^{-\frac{1+d}{2}}I\left\{x\geq a\right\}\} \end{array}$$
  for −∞ < *x* < +∞, −∞ < *a* < +∞, *b* > 0, *c* > 0 and *d* > 0. The contributed R package gamlss.dist due to [49] is used to compute this PDF *g*.
- Skew *t* distribution of type 5 [52]: g = “ST5”, starts = c(a, b, c, d) and
  $$\begin{array}{c} g\left(x\right)=\frac{1}{b2^{\alpha +\beta -1}\sqrt{\alpha +\beta }B\left(\alpha ,\beta \right)}{\left[1+\frac{x-a}{b\sqrt{\alpha +\beta +{\left(\frac{x-a}{b}\right)}^{2}}}\right]}^{\alpha +\frac{1}{2}}\\ \cdot {\left[1-\frac{x-a}{b\sqrt{\alpha +\beta +{\left(\frac{x-a}{b}\right)}^{2}}}\right]}^{\beta +\frac{1}{2}} \end{array}$$
  for −∞ < *x* < +∞, −∞ < *a* < +∞, *b* > 0, −∞ < *c* < +∞ and *d* > 0, where $c=\frac{\alpha -\beta }{\sqrt{\alpha \beta (\alpha +\beta )}}$ and $d=\frac{2}{\alpha +\beta }$. The contributed R package gamlss.dist due to [49] is used to compute this PDF *g*.
- Sinh arcsinh distribution [53]: g = “SHASH”, starts = c(a, b, c, d) and
  $$\begin{array}{c} g\left(x\right)=\frac{\beta \exp(-\alpha^{2}/2)}{\sqrt{2\pi }\sqrt{b^{2}+{\left(x-a\right)}^{2}}} \end{array}$$
  for −∞ < *x* < +∞, −∞ < *a* < +∞, *b* > 0, *c* > 0 and *d* > 0, where
  $$\begin{array}{c} \alpha =\frac{1}{2}\{\exp[d\;\text{arcsinh}(\frac{x-a}{b})]-\exp[-c\;\text{arcsinh}(\frac{x-a}{b})]\} \end{array}$$
  and
  $$\begin{array}{c} \beta =\frac{1}{2}\{d\exp[d\;\text{arcsinh}(\frac{x-a}{b})]+c\exp[-c\;\text{arcsinh}(\frac{x-a}{b})]\}. \end{array}$$
  The contributed R package gamlss.dist due to [49] is used to compute this PDF *g*.
- Sinh arcsinh distribution [54]: g = “SHASHo”, starts = c(a, b, c, d) and
  $$\begin{array}{c} g\left(x\right)=\frac{\beta d\exp(-\alpha^{2}/2)}{2\sqrt{2\pi }\sqrt{b^{2}+{\left(x-a\right)}^{2}}} \end{array}$$
  for −∞ < *x* < +∞, −∞ < *a* < +∞, *b* > 0, −∞ < *c* < +∞ and *d* > 0, where
  $$\begin{array}{c} \alpha =\sinh[d\;\text{arcsin}(\frac{x-a}{b})-c] \end{array}$$
  and
  $$\begin{array}{c} \beta =\cosh[d\;\text{arcsin}(\frac{x-a}{b})-c]. \end{array}$$
  The contributed R package gamlss.dist due to [49] is used to compute this PDF *g*.
- Sinh arcsinh distribution [54]: g = “SHASH2”, starts = c(a, b, c, d) and
  $$\begin{array}{c} g\left(x\right)=\frac{\beta d^{2}}{\sqrt{2\pi }\sqrt{b^{2}d^{2}+{\left(x-a\right)}^{2}}}-\exp(-\frac{\alpha^{2}}{2}) \end{array}$$
  for −∞ < *x* < +∞, −∞ < *a* < +∞, *b* > 0, −∞ < *c* < +∞ and *d* > 0, where
  $$\begin{array}{c} \alpha =\sinh[d\;\text{arcsin}(\frac{x-a}{bd})-c] \end{array}$$
  and
  $$\begin{array}{c} \beta =\cosh[d\;\text{arcsin}(\frac{x-a}{bd})-c]. \end{array}$$
  The contributed R package gamlss.dist due to [49] is used to compute this PDF *g*.
- Exponential generalized beta type 2 distribution [55]: g = “EGB2”, starts = c(a, b, c, d) and
  $$\begin{array}{c} g\left(x\right)=\frac{1}{|b|B\left(c,d\right)}\text{exp}\left(c\frac{x-a}{b}\right){\left[1+\text{exp}\left(\frac{x-a}{b}\right)\right]}^{-c-d} \end{array}$$
  for −∞ < *x* < +∞, −∞ < *a* < +∞, −∞ < *b* < +∞, *c* > 0 and *d* > 0. The contributed R package gamlss.dist due to [49] is used to compute this PDF *g*.
- Johnson’s *S*_*u*_ distribution [56]: g = “JSU”, starts = c(a, b, c, d) and
  $$\begin{array}{c} g\left(x\right)=\frac{1}{\sqrt{2\pi }\alpha bd}\frac{\exp(-r^{2}/2)}{\sqrt{1+z^{2}}} \end{array}$$
  for −∞ < *x* < +∞, −∞ < *a* < +∞, *b* > 0, −∞ < *c* < +∞ and *d* > 0, where $r=-c+\frac{1}{d}\;\text{arcsinh}\left(z\right)$, $z=\frac{x-a}{\alpha b}-\sqrt{w}\sinh\Omega$, $\alpha =\sqrt{\frac{w-1}{2}}\sqrt{1+w\cosh(2\Omega )}$, *w* = exp (*d*^2^) and Ω = −*cd*. The contributed R package gamlss.dist due to [49] is used to compute this PDF *g*.
- Johnson’s *S*_*u*_ distribution [56]: g = “JSUo”, starts = c(a, b, c, d) and
  $$\begin{array}{c} g\left(x\right)=\frac{d}{\sqrt{2\pi }\sqrt{b^{2}+{\left(x-a\right)}^{2}}}\text{exp}\left\{-\frac{1}{2}{\left[c+d\;\text{arcsinh}\left(\frac{x-a}{b}\right)\right]}^{2}\right\} \end{array}$$
  for −∞ < *x* < +∞, −∞ < *a* < +∞, *b* > 0, −∞ < *c* < +∞ and *d* > 0. The contributed R package gamlss.dist due to [49] is used to compute this PDF *g*.
- Skew generalized *t* distribution [57]: g = “sgt”, starts = c(a, b, c, d, e) and
  $$\begin{array}{c} g\left(x\right)=\frac{d}{2\nu be^{\frac{1}{d}}B\left(\frac{1}{d},e\right)}{\left\{1+\frac{{\left|x-b+m\right|}^{d}}{e{\left(\nu b\right)}^{d}{\left[c\;\text{sign}\left(x-b+m\right)+1\right]}^{d}}\right\}}^{-\frac{1}{d}-c} \end{array}$$
  for −∞ < *x* < +∞, −∞ < *a* < +∞, *b* > 0, −1 < *c* < 1, *d* > 0 and *e* > 0, where
  $$\begin{array}{c} m=\frac{2\nu bce^{\frac{1}{d}}B(\frac{2}{d},e-\frac{1}{d})}{B(\frac{1}{d},e)} \end{array}$$
  and
  $$\begin{array}{c} \nu =e^{-\frac{1}{d}}{\left[\left(3c^{2}+1\right)\frac{B\left(\frac{3}{d},e-\frac{3}{d}\right)}{B\left(\frac{1}{d},e\right)}-4c^{2}\frac{B^{2}\left(\frac{2}{d},e-\frac{1}{d}\right)}{B^{2}\left(\frac{1}{d},e\right)}\right]}^{-1/2}. \end{array}$$
  The contributed R package sgt due to [58] is used to compute this PDF *g*.
- Skew hyperbolic distribution [59]: g = “skewhyp”, starts = c(a, b, c, d) and
  $$\begin{array}{c} g\left(x\right)=\frac{2^{\frac{1-d}{2}}b^{d}|c{|}^{\frac{1+d}{2}}\text{exp}\left[c\left(x-a\right)\right]K_{\frac{1+d}{2}}\left(|c|\sqrt{b^{2}+{\left(x-a\right)}^{2}}\right)}{\sqrt{\pi }\Gamma \left(\frac{d}{2}\right){\left[b^{2}+{\left(x-a\right)}^{2}\right]}^{\frac{1+d}{4}}} \end{array}$$
  for −∞ < *x* < +∞, −∞ < *a* < +∞, *b* > 0, −∞ < *c* < +∞ and *d* > 0, where *K*_*ν*_(⋅) denotes the modified Bessel function of the second kind of order *ν* defined by
  $$\begin{array}{c} K_{\nu }\left(x\right)=\{\begin{array}{cc} \frac{\pi \text{csc}(\pi \nu )}{2}[I_{-\nu }\left(x\right)-I_{\nu }\left(x\right)], & \text{if}\;\nu \notin Z\text{,}\\ \lim_{\mu \to \nu }K_{\mu }\left(x\right), & \text{if}\;\nu \in Z\text{,} \end{array} \end{array}$$
  where *I*_*ν*_(⋅) denotes the modified Bessel function of the first kind of order *ν* defined by
  $$\begin{array}{c} I_{\nu }\left(x\right)={\sum_{k=0}^{\infty }\frac{1}{\Gamma \left(k+\nu +1\right)k!}\left(\frac{x}{2}\right)}^{2k+\nu }. \end{array}$$
  The contributed R package SkewHyperbolic due to [60] is used to compute this PDF *g*.
- Asymmetric Laplace distribution [61]: g = “asl”, starts = c(a, c, b) and
  $$\begin{array}{c} g\left(x\right)=\{\begin{array}{c} \frac{\sqrt{2}}{b}\frac{\kappa }{1+\kappa^{2}}\exp(-\frac{\sqrt{2}\kappa }{b}|x-a|),\text{if}\;x\geq a\text{,}\\ \frac{\sqrt{2}}{b}\frac{\kappa }{1+\kappa^{2}}\exp(-\frac{\sqrt{2}}{b\kappa }|x-a|),\text{if}\;x<a \end{array} \end{array}$$
  for −∞ < *x* < +∞, −∞ < *a* < +∞, *b* > 0 and −∞ < *c* < +∞, where $\kappa =(\sqrt{2b^{2}+c^{2}}-c)/(\sqrt{2}b)$. The contributed R package cubfits due to [62] is used to compute this PDF *g*.
- Asymmetric Laplace distribution [61]: g = “asla”, starts = c(a, c, b) and
  $$\begin{array}{c} g\left(x\right)=\{\begin{array}{c} \frac{\sqrt{2}}{b}\frac{c}{1+c^{2}}\exp(-\frac{\sqrt{2}c}{b}|x-a|),\text{if}\;x\geq a\text{,}\\ \frac{\sqrt{2}}{b}\frac{c}{1+c^{2}}\exp(-\frac{\sqrt{2}}{bc}|x-a|),\text{if}\;x<a \end{array} \end{array}$$
  for −∞ < *x* < +∞, −∞ < *a* < +∞, *b* > 0 and *c* > 0. The contributed R package cubfits due to [62] is used to compute this PDF *g*.
- Asymmetric Laplace distribution [63]: g = “al”, starts = c(a, b, c) and
  $$\begin{array}{c} g\left(x\right)=\frac{c\left(1-c\right)}{b}\text{exp}\left[-\frac{x-a}{b}(c-I\left\{x<a\right\})\right] \end{array}$$
  for −∞ < *x* < +∞, −∞ < *a* < +∞, *b* > 0 and 0 < *c* < 1. The contributed R package lqmm due to [64] is used to compute this PDF *g*.
- Polynomial tail Laplace distribution: g = “PTL”, starts = c(a, b, c) and
  $$\begin{array}{c} g\left(x\right)=\{\begin{array}{c} \frac{a(\frac{x^{2}}{2}+2x+2)+b[\exp(\frac{x}{b})-\exp(-\frac{2}{b})]+c(\frac{x^{3}}{3}+4x+\frac{16}{3})}{4a+2b[1-\exp(-\frac{2}{b})]+\frac{32c}{3}},\\ \;\text{if}\;-2\leq x\leq 0\text{,}\\ \frac{a(2x-\frac{x^{2}}{2}-2)+b[\exp(-\frac{2}{b})-\exp(\frac{x}{b})]+c(4x-\frac{x^{3}}{3}-\frac{16}{3})}{4a+2b[1-\exp(-\frac{2}{b})]+\frac{32c}{3}},\\ \;\text{if}\;0<x\leq 2 \end{array} \end{array}$$
  for −∞ < *x* < +∞, *a* > 0, *b* > 0 and *c* > 0. The contributed R package LCA due to [65] is used to compute this PDF *g*.
- Generalized asymmetric *t* distribution [66]: g = “gat”, starts = c(a, b, c, d, e) and
  $$\begin{array}{c} g\left(x\right)=\frac{d}{b\left(\frac{1}{e}+e\right)c^{\frac{1}{d}}B\left(\frac{1}{d},c\right)}\{\begin{array}{cc} {\left\{1+\frac{{\left[-\frac{\left(x-a\right)e}{b}\right]}^{d}}{c}\right\}}^{-c-\frac{1}{d}}, & \text{if}\;x<a\text{,}\\ {\left\{1+\frac{{\left[-\frac{x-a}{be}\right]}^{d}}{c}\right\}}^{-c-\frac{1}{d}}, & \text{if}\;x\geq a \end{array} \end{array}$$
  for −∞ < *x* < +∞, −∞ < *a* < +∞, *b* > 0, *c* > 0, *d* > 0 and *e* > 0. The contributed R package GEVStableGarch due to [67] is used to compute this PDF *g*.
- Variance gamma distribution [68]: g = “vg”, starts = c(a, b, c, d) and
  $$\begin{array}{ccc} g\left(x\right) & = & \frac{2}{\sqrt{2\pi }bd^{\frac{1}{d}}\Gamma \left(1/d\right)}{\left(\frac{2b^{2}}{d}+c^{2}\right)}^{\frac{1}{d}-\frac{1}{4}}{\left|x-a\right|}^{\frac{1}{d}-\frac{1}{2}}\\ & & \cdot \text{exp}\left[\frac{c{\left(x-a\right)}^{2}}{b^{2}}\right]K_{\frac{1}{d}-\frac{1}{2}}\left(\sqrt{\frac{2b^{2}}{d}+c^{2}}\frac{\left|x-a\right|}{b^{2}}\right) \end{array}$$
  for −∞ < *x* < +∞, −∞ < *a* < +∞, *b* > 0, −∞ < *c* < +∞ and *d* > 0. The contributed R package VarianceGamma due to [69] is used to compute this PDF *g*.
- Normal inverse Gaussian distribution [68]: g = “nig”, starts = c(a, b, c, d) and
  $$\begin{array}{c} g\left(x\right)=\frac{bc}{\pi \sqrt{b^{2}+{\left(x-a\right)}^{2}}}\exp[b\sqrt{c^{2}-d^{2}}+d\left(x-a\right)]K_{1}(c\sqrt{b^{2}+{\left(x-a\right)}^{2}}) \end{array}$$
  for −∞ < *x* < +∞, −∞ < *a* < +∞, *b* > 0, *c* > 0 and −∞ < *d* < +∞. The contributed R package GeneralizedHyperbolic due to [70] is used to compute this PDF *g*.
- Hyperbolic distribution [68]: g = “hyperb”, starts = c(a, b, c, d) and
  $$\begin{array}{c} g\left(x\right)=\frac{\sqrt{c^{2}-d^{2}}}{2bcK_{1}(b\sqrt{c^{2}-d^{2}})}\exp[-c\sqrt{b^{2}+{\left(x-a\right)}^{2}}+d\left(x-a\right)] \end{array}$$
  for −∞ < *x* < +∞, −∞ < *a* < +∞, *b* > 0, *c* > 0 and −∞ < *d* < +∞. The contributed R package GeneralizedHyperbolic due to [70] is used to compute this PDF *g*.
- Skew Laplace distribution [71]: g = “skewlap”, starts = c(a, b, c) and
  $$\begin{array}{c} g\left(x\right)=\{\begin{array}{c} \frac{1}{b+c}\exp(\frac{x-a}{b}),\text{if}\;x\leq a\text{,}\\ \frac{1}{b+c}\exp(-\frac{x-a}{c}),\text{if}\;x>a \end{array} \end{array}$$
  for −∞ < *x* < +∞, −∞ < *a* < +∞, *b* > 0 and *c* > 0. The contributed R package GeneralizedHyperbolic due to [70] is used to compute this PDF *g*.
- Slash distribution [72]: g = “slash”, starts = c(a, b) and
  $$\begin{array}{c} g\left(x\right)=1-\frac{b}{\sqrt{2\pi }{\left(x-a\right)}^{2}}\exp[-\frac{{\left(x-a\right)}^{2}}{2b^{2}}] \end{array}$$
  for −∞ < *x* < +∞, −∞ < *a* < +∞ and *b* > 0. The contributed R package VGAM due to [73] is used to compute this PDF *g*.
- Beta normal distribution [74]: g = “betanorm”, starts = c(a, b, c, d) and
  $$\begin{array}{c} g\left(x\right)=\frac{1}{bB\left(c,d\right)}\phi \left(\frac{x-a}{b}\right){\left[\Phi \left(\frac{x-a}{b}\right)\right]}^{c-1}{\left[\Phi \left(\frac{a-x}{b}\right)\right]}^{d-1} \end{array}$$
  for −∞ < *x* < +∞, −∞ < *a* < +∞, *b* > 0, *c* > 0 and *d* > 0. The contributed R package VGAM due to [73] is used to compute this PDF *g*.
- Laplace distribution: g = “laplace”, starts = c(a, b) and
  $$\begin{array}{c} g\left(x\right)=\frac{1}{2b}\exp(-\frac{|x-a|}{b}) \end{array}$$
  for −∞ < *x* < +∞, −∞ < *a* < +∞ and *b* > 0. The contributed R package VGAM due to [73] is used to compute this PDF *g*.
- Short tailed symmetric distribution [75]: g = “tikuv”, starts = c(a, b, c) and
  $$\begin{array}{c} g\left(x\right)=\frac{C}{\sqrt{2\pi }b}\phi {\left[1+\frac{{\left(x-a\right)}^{2}}{2cb^{2}}\right]}^{2}\text{exp}\left[-\frac{{\left(x-a\right)}^{2}}{2b^{2}}\right] \end{array}$$
  for −∞ < *x* < +∞, −∞ < *a* < +∞, *b* > 0 and *c* > 0, where *C* denotes the normalizing constant. The contributed R package VGAM due to [73] is used to compute this PDF *g*.
- Log gamma distribution [76]: g = “lgamma”, starts = c(a) and
  $$\begin{array}{c} g\left(x\right)=\frac{|a|a^{-2a^{-2}}}{\Gamma \left(a^{-2}\right)}\text{exp}\left\{-a^{-2}\left[ax-\text{exp}\left(ax\right)\right]\right\} \end{array}$$
  for −∞ < *x* < +∞ and −∞ < *a* < +∞. The contributed R package ordinal due to [77] is used to compute this PDF *g*.

mwrappedg will output the following: parameter estimates, standard errors, 95 percent confidence intervals, value of Akaike Information Criterion, value of Consistent Akaike Information Criterion, value of Bayesian Information Criterion, value of Hannan Quinn Information Criterion, Cramer von Misses statistic value, Anderson Darling statistic value, minimum value of the negative log likelihood, Kolmogorov Smirnov statistic value, its *p* value and convergence status of the minimization of the negative log likelihood. These were computed using the R package AdequacyModel due to [78]. There are other packages for fitting univariate distributions, for example, the R package fitdistrplus due to [79]. But none of these packages give as much output as [78] gives.

plotfour draws four plots of the PDF, four plots of the CDF, four plots of the quantile function or four histograms of the radii of 100 random numbers of a specified wrapped distribution. The wrapped distribution must be specified by g and K as explained before. plotit is a character string saying what is to be plotted. It should take one of “pdf”, “cdf”, “quantile” or “random”. para is a list with four components, each component is a vector specifying the parameter values of the chosen wrapped distribution.

## Illustrations

Here, we provide several illustrations of the practical use of the package Wrapped.

The first illustration plots the PDFs of the wrapped beta normal, wrapped skew normal, wrapped asymmetric Laplace and wrapped skew *t* type 3 distributions for selected parameter values.

plotfour(“betanorm”,K = 100,para = list(c(0,1,0.1,9),c(0,1,0.1,20),

c(0,1,20,0.1),c(0,1,3,3)), plotit = “pdf”)

plotfour(“sn”,K = 100,para = list(c(0,1,-2),c(0,1,0),

c(0,1,2),c(0,1,10)), plotit = “pdf”)

plotfour(“asla”,K = 100,para = list(c(0,1,1),c(0,2,1),

c(0,5,1),c(0,50,1)), plotit = “pdf”)

plotfour(“ST3”,K = 100,para = list(c(0,1,1,1),c(0,1,2,5),

c(0,1,0.1,5),c(0,1,50,50)), plotit = “pdf”)

**Fig 1:**
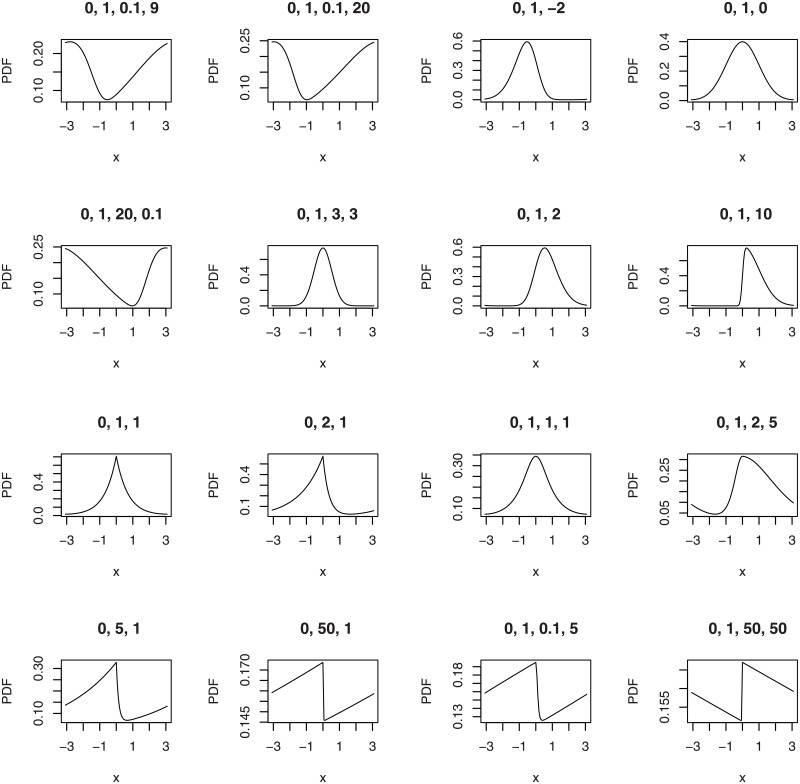
PDFs of the wrapped beta normal (four plots in the top left), wrapped skew normal (four plots in the top right) wrapped asymmetric Laplace (four plots in the bottom left) and wrapped skew *t* type 3 (four plots in the bottom right) distributions for selected parameter values.

The plots generated are collated in Fig 1.

The second illustration plots the CDFs of the wrapped beta normal, wrapped skew normal, wrapped asymmetric Laplace and wrapped skew *t* type 3 distributions for selected parameter values.

plotfour(“betanorm”,K = 100,para = list(c(0,1,0.1,9),c(0,1,0.1,20),

c(0,1,20,0.1),c(0,1,3,3)), plotit = “cdf”)

plotfour(“sn”,K = 100,para = list(c(0,1,-2),c(0,1,0),

c(0,1,2),c(0,1,10)), plotit = “cdf”)

plotfour(“asla”,K = 100,para = list(c(0,1,1),c(0,2,1),

c(0,5,1),c(0,50,1)), plotit = “cdf”)

plotfour(“ST3”,K = 100,para = list(c(0,1,1,1),c(0,1,2,5),

c(0,1,0.1,5),c(0,1,50,50)), plotit = “cdf”)

**Fig 2:**
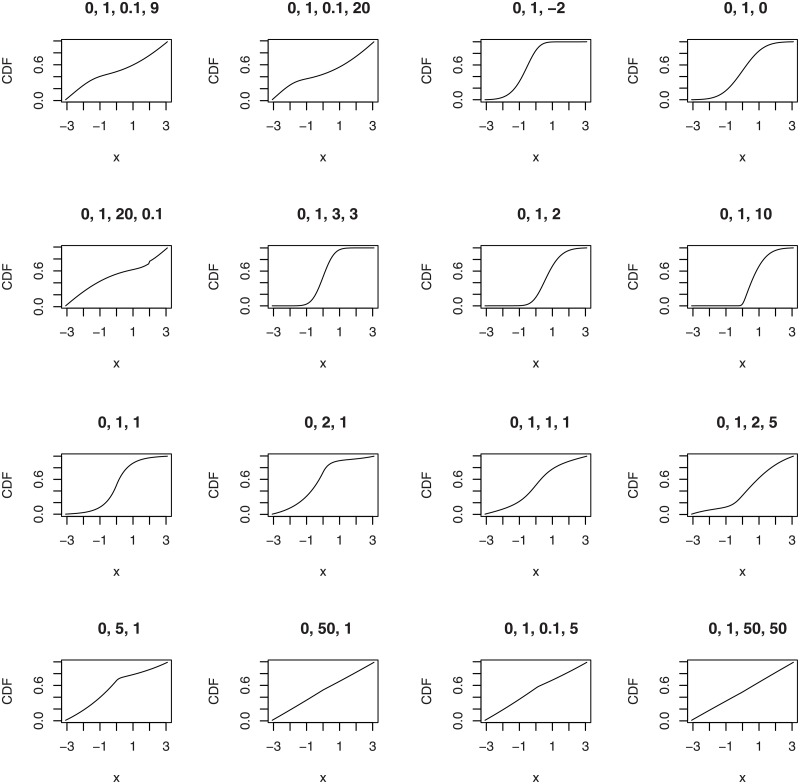
CDFs of the wrapped beta normal (four plots in the top left), wrapped skew normal (four plots in the top right) wrapped asymmetric Laplace (four plots in the bottom left) and wrapped skew *t* type 3 (four plots in the bottom right) distributions for selected parameter values.

The plots generated are collated in Fig 2.

The third illustration plots the quantile functions of the wrapped beta normal, wrapped skew normal, wrapped asymmetric Laplace and wrapped skew *t* type 3 distributions for selected parameter values.

plotfour(“betanorm”,K = 100,para = list(c(0,1,0.1,9),c(0,1,0.1,20),

c(0,1,20,0.1),c(0,1,3,3)), plotit = “quantile”)

plotfour(“sn”,K = 100,para = list(c(0,1,-2),c(0,1,0),

c(0,1,2),c(0,1,10)), plotit = “quantile”)

plotfour(“asla”,K = 100,para = list(c(0,1,1),c(0,2,1),

c(0,5,1),c(0,50,1)), plotit = “quantile”)

plotfour(“ST3”,K = 100,para = list(c(0,1,1,1),c(0,1,2,5),

c(0,1,0.1,5),c(0,1,50,50)), plotit = “quantile”)

**Fig 3:**
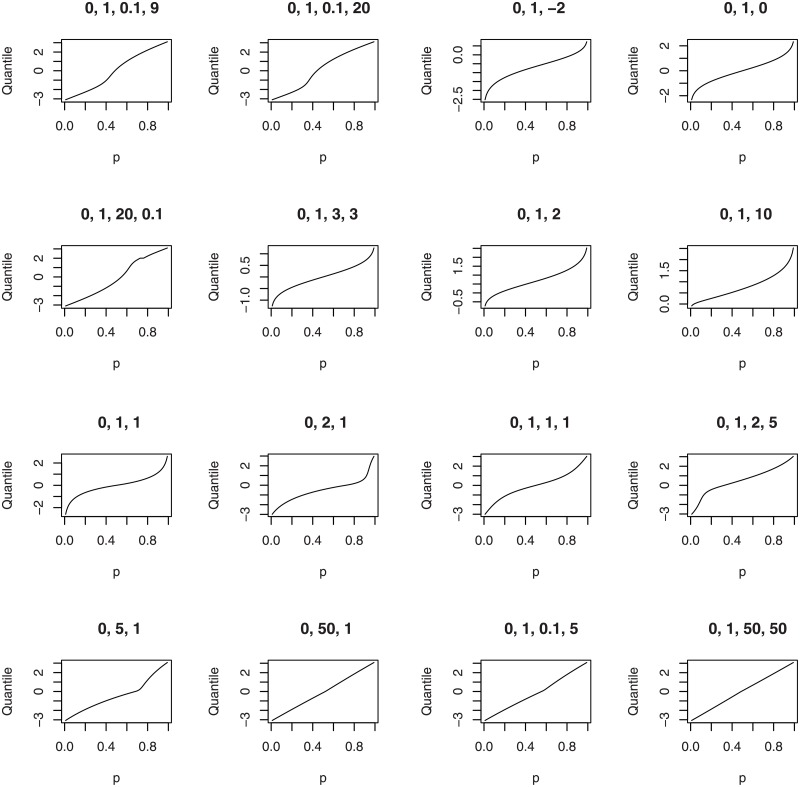
Qunatile functions of the wrapped beta normal (four plots in the top left), wrapped skew normal (four plots in the top right) wrapped asymmetric Laplace (four plots in the bottom left) and wrapped skew *t* type 3 (four plots in the bottom right) distributions for selected parameter values.

The plots generated are collated in Fig 3.

The fourth illustration plots the histograms of the radii of 100 random numbers generated from the wrapped beta normal, wrapped skew normal, wrapped asymmetric Laplace and wrapped skew *t* type 3 distributions for selected parameter values.

plotfour(“betanorm”,K = 100,para = list(c(0,1,0.1,9),c(0,1,0.1,20),

c(0,1,20,0.1),c(0,1,3,3)), plotit = “random”)

plotfour(“sn”,K = 100,para = list(c(0,1,-2),c(0,1,0),

c(0,1,2),c(0,1,10)), plotit = “random”)

plotfour(“asla”,K = 100,para = list(c(0,1,1),c(0,2,1),

c(0,5,1),c(0,50,1)), plotit = “random”)

plotfour(“ST3”,K = 100,para = list(c(0,1,1,1),c(0,1,2,5),

c(0,1,0.1,5),c(0,1,50,50)), plotit = “random”)

**Fig 4:**
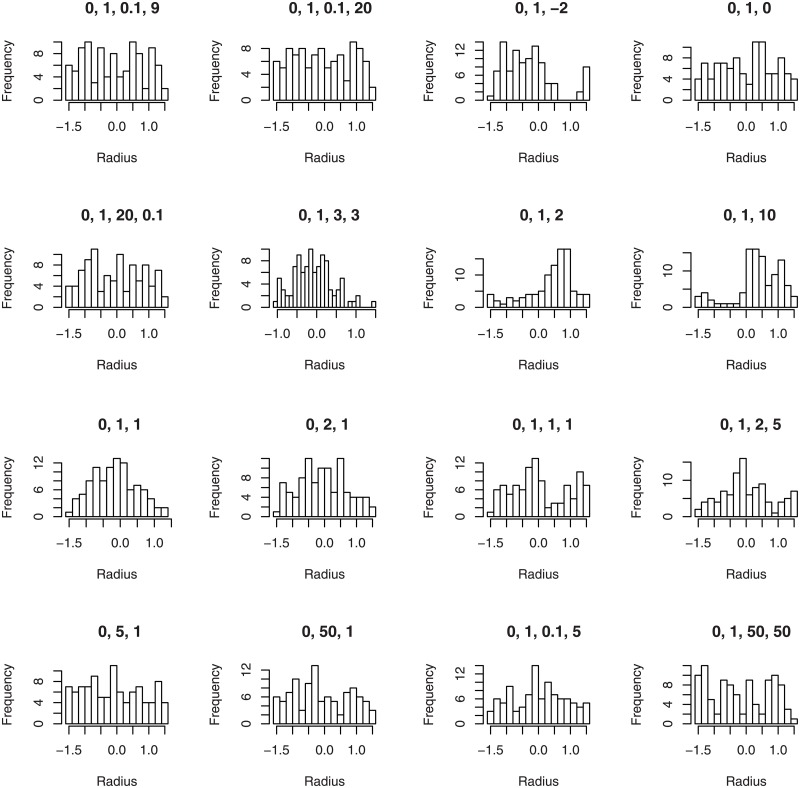
Histograms of 100 random numbers generated from the wrapped beta normal (four plots in the top left), wrapped skew normal (four plots in the top right) wrapped asymmetric Laplace (four plots in the bottom left) and wrapped skew *t* type 3 (four plots in the bottom right) distributions for selected parameter values.

The plots generated are collated in Fig 4.

We see that a variety of symmetric and asymmetric shapes are possible for the PDFs, CDFs, quantile functions and histograms.

The following code shows how to use Wrapped to fit wrapped normal, wrapped logistic, wrapped Gumbel, wrapped Laplace and wrapped Student’s *t* distributions to data. The data used are 30 cross-beds azimuths of palaeocurrents from [80].

x = c(294, 177, 257, 301, 257, 267, 329, 177, 241, 315,

229, 239, 277, 250, 287, 281, 166, 229, 254, 232,

290, 245, 245, 214, 272, 224, 215, 242, 186, 224)

x = 2 * pi * x / 360 – pi

mwrappedg(“norm”, data = x, starts = c(mean(x), 1),

K = 100, method = “BFGS”)

mwrappedg(“logis”, data = x, starts = c(mean(x), 1),

K = 100, method = “BFGS”)

mwrappedg(“gumbel”, data = x, starts = c(mean(x), 1),

K = 100, method = “BFGS”)

mwrappedg(“laplace”, data = x, starts = c(mean(x), 1),

K = 100, method = “BFGS”)

mwrappedg(“t.scaled”, data = x, starts = c(mean(x), sd(x), 50),

K = 100, method = “BFGS”)

For each of the five fitted wrapped distributions, the output gives the parameter estimates, standard errors, 95 percent confidence intervals, value of Akaike Information Criterion, value of Consistent Akaike Information Criterion, value of Bayesian Information Criterion, value of Hannan Quinn Information Criterion, Cramer von Misses statistic value, Anderson Darling statistic value, minimum value of the negative log likelihood, Kolmogorov Smirnov statistic value, its *p* value and convergence status of the minimization of the negative log likelihood.

The output for the fitted wrapped normal distribution is as follows.

$$\begin{array}{c} \begin{array}{rrrrr} \$\text{Estimates} & & & & \\ & \text{MLE} & \text{Std}.\text{Dev}. & \text{Inf}.95\%\;\text{CI} & \text{Sup}.95\%\;\text{CI}\\ \left[1,\right] & 1.1728617 & 0.12682025 & 0.9242986 & 1.421425\\ \left[2,\right] & 0.6946231 & 0.08967455 & 0.5188642 & 0.870382 \end{array}\\ \begin{array}{l} \begin{array}{rrrrr} \$\text{Measures} & & & & \\ \text{AIC} & \text{CAIC} & \text{BIC} & \text{HQIC} & \text{W}\\ 67.27319 & 67.71764 & 70.07559 & 68.1697 & 0.02754482 \end{array}\\ \;\text{A}\;\text{Min}(-\text{log}(\text{Likelihood}))\\ \begin{array}{rr} 0.2095757 & \;31.6366 \end{array} \end{array}\\ \begin{array}{l} \$`\text{Kolmogorov}-\text{Smirnov}\;\text{Test}`\\ \begin{array}{rr} \text{KS}\;\text{Statistic} & \text{KS}\;\text{p-value}\\ 0.08226683 & 0.9872238 \end{array} \end{array}\\ \$`\text{Convergence}\;\text{Status}`\\ "\text{Algorithm}\;\text{Converged}" \end{array}$$

The output for the fitted wrapped logistic distribution is as follows.

$$\begin{array}{c} \begin{array}{rrrrr} \$\text{Estimates} & & & & \\ & \text{MLE} & \text{Std}.\text{Dev}. & \text{Inf}.95\%\;\text{CI} & \text{Sup}.95\%\;\text{CI}\\ \left[1,\right] & 1.181547 & 0.12672650 & 0.9331680 & 1.4299267\\ \left[2,\right] & 0.398243 & 0.06068586 & 0.2793009 & 0.5171851 \end{array}\\ \begin{array}{l} \begin{array}{rrrrr} \$\text{Measures} & & & & \\ \text{AIC} & \text{CAIC} & \text{BIC} & \text{HQIC} & \text{W}\\ 68.10648 & 68.55092 & 70.90887 & 69.00299 & 0.02303805 \end{array}\\ \;\text{A}\;\text{Min}(-\text{log}(\text{Likelihood}))\\ \begin{array}{rr} 0.1835242 & \;32.05324 \end{array} \end{array}\\ \begin{array}{l} \$`\text{Kolmogorov}-\text{Smirnov}\;\text{Test}`\\ \begin{array}{rr} \text{KS}\;\text{Statistic} & \text{KS}\;\text{p-value}\\ 0.06864052 & 0.9989203 \end{array} \end{array}\\ \$`\text{Convergence}\;\text{Status}`\\ "\text{Algorithm}\;\text{Converged}" \end{array}$$

The output for the fitted wrapped Gumbel distribution is as follows.

$$\begin{array}{c} \begin{array}{rrrrr} \$\text{Estimates} & & & & \\ & \text{MLE} & \text{Std}.\text{Dev}. & \text{Inf}.95\%\;\text{CI} & \text{Sup}.95\%\;\text{CI}\\ \left[1,\right] & 1.8245372 & 0.13432556 & 0.5612639 & 1.0878105\\ \left[2,\right] & 0.6860317 & 0.09273898 & 0.5042667 & 0.8677968 \end{array}\\ \begin{array}{l} \begin{array}{rrrrr} \$\text{Measures} & & & & \\ \text{AIC} & \text{CAIC} & \text{BIC} & \text{HQIC} & \text{W}\\ 71.98751 & 72.43195 & 74.7899 & 72.88402 & 0.04632486 \end{array}\\ \text{A}\;\text{Min}(-\text{log}(\text{Likelihood}))\\ \begin{array}{rr} 0.380803 & \;33.99375 \end{array} \end{array}\\ \begin{array}{l} \$`\text{Kolmogorov}-\text{Smirnov}\;\text{Test}`\\ \begin{array}{rr} \text{KS}\;\text{Statistic} & \text{KS}\;\text{p-value}\\ 0.1710141 & 0.3441205 \end{array} \end{array}\\ \$`\text{Convergence}\;\text{Status}`\\ "\text{Algorithm}\;\text{Converged}" \end{array}$$

The output for the fitted wrapped Laplace distribution is as follows.

$$\begin{array}{c} \begin{array}{rrrrr} \$\text{Estimates} & & & & \\ & \text{MLE} & \text{Std}.\text{Dev}. & \text{Inf}.95\%\;\text{CI} & \text{Sup}.95\%\;\text{CI}\\ \left[1,\right] & 1.1344640 & 0.01651829 & 1.1020887 & 1.1668392\\ \left[2,\right] & 0.5456715 & 0.10041207 & 0.3488675 & 0.7424756 \end{array}\\ \begin{array}{l} \begin{array}{rrrrr} \$\text{Measures} & & & & \\ \text{AIC} & \text{CAIC} & \text{BIC} & \text{HQIC} & \text{W}\\ 69.10345 & 69.5479 & 71.90585 & 69.99996 & 0.02765046 \end{array}\\ \text{A}\;\text{Min}(-\text{log}(\text{Likelihood}))\\ \begin{array}{rr} 0.2146212 & \;32.55173 \end{array} \end{array}\\ \begin{array}{l} \$`\text{Kolmogorov}-\text{Smirnov}\;\text{Test}`\\ \begin{array}{rr} \text{KS}\;\text{Statistic} & \text{KS}\;\text{p-value}\\ 0.1016237 & 0.9160182 \end{array} \end{array}\\ \$`\text{Convergence}\;\text{Status}`\\ "\text{Algorithm}\;\text{Converged}" \end{array}$$

The output for the fitted wrapped Student’s *t* distribution is as follows.

$$\begin{array}{c} \begin{array}{rrrrr} \$\text{Estimates} & & & & \\ & \text{MLE} & \text{Std}.\text{Dev}. & \text{Inf}.95\%\text{CI} & \text{Sup}.95\%\text{CI}\\ \left[1,\right] & 1.1740348 & 0.12720525 & 0.9247171 & 1.4233525\\ \left[2,\right] & 0.6864103 & 0.09429592 & 0.5015937 & 0.8712269\\ \left[3,\right] & 67.5447148 & 224.76746687 & -372.9914252 & 508.0808548 \end{array}\\ \begin{array}{l} \begin{array}{rrrrr} \$\text{Measures} & & & & \\ \text{AIC} & \text{CAIC} & \text{BIC} & \text{HQIC} & \text{W}\\ 69.36032 & 70.2834 & 73.56391 & 70.70509 & 0.02669693 \end{array}\\ \text{A}\;\text{Min}(-\text{log}(\text{Likelihood}))\\ \begin{array}{rr} 0.2049471 & \;31.68016 \end{array} \end{array}\\ \begin{array}{l} \$`\text{Kolmogorov}-\text{Smirnov}\;\text{Test}`\\ \begin{array}{rr} \text{KS}\;\text{Statistic} & \text{KS}\;\text{p-value}\\ 0.0808072 & 0.9895772 \end{array} \end{array}\\ \$`\text{Convergence}\;\text{Status}`\\ "\text{Algorithm}\;\text{Converged}" \end{array}$$

The standard errors appear small compared to the parameter estimates for each fitted distribution. An exception is the Student’s *t* wrapped distribution. Also the *p*-value for each fitted distribution appears acceptable at the five percent significance level.

The wrapped normal distribution gives the smallest values for the Cramer von Misses statistic, Anderson Darling statistic, Akaike Information Criterion, Consistent Akaike Information Criterion, Bayesian Information Criterion, Hannan Quinn information criterion and the minimum of the negative log likelihood function. But the wrapped logistic distribution gives the smallest Kolmogorov Smirnov test statistic and the largest *p*-value.

**Fig 5:**
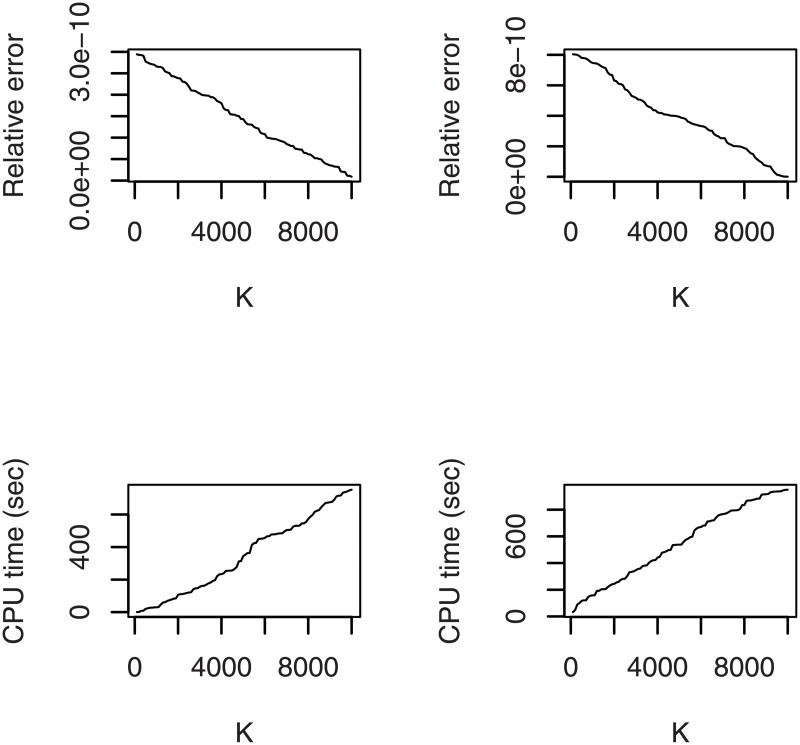
Relative error of the approximate PDF (top left), and CDF (top right). Central processing unit time for 100 computations of the approximate PDF (bottom left), and CDF (right left).

The computations of the PDFs, CDFs and quantile functions are based on the approximate PDF and CDF. We now check to the goodness of these approximations and recommend a value for *K*. Fig 5 plots the relative errors of the approximate PDF and CDF versus *K* = 100, 200,…,10000. It also plots the central processing unit time for 100 computations of the approximate PDF and CDF versus *K* = 100, 200,…,10000. The relative errors for the approximate PDF and CDF were computed as

$$\begin{array}{c} {\left[\sum_{k=-K}^{K}g\left(x+2\pi k\right)\right]}^{-1}\left|\sum_{k=-K}^{K}g\left(x+2\pi k\right)-\sum_{k=-K-1}^{K+1}g\left(x+2\pi k\right)\right| \end{array}$$

and

$$\begin{array}{ccc} & & {\left\{\sum_{k=-K}^{K}\left[G\left(x+2\pi k\right)-G\left(-\pi +2\pi k\right)\right]\right\}}^{-1}|\sum_{k=-K}^{K}\left[G\left(x+2\pi k\right)-G\left(-\pi +2\pi k\right)\right]\\ & & \;-\sum_{k=-K-1}^{K+1}\left[G\left(x+2\pi k\right)-G\left(-\pi +2\pi k\right)\right]|, \end{array}$$

respectively.

We see that the order of the relative errors is 10^−10^ when *K* = 100. Thereafter it decreases approximately linearly. The central processing unit times are less than 10 seconds when *K* = 100. Thereafter they increase approximately linearly.

In Fig 5, we have taken *g* and *G* to correspond to the beta normal wrapped distribution with a = 0, b = 1, c = 0.1 and d = 0.9. The figures were similar for other wrapped distributions and a wide range of parameter values. In particular, the order of the relative errors when *K* = 100 was always 10^−10^ and the central processing unit times when *K* = 100 were always less than 10 seconds. Furthermore, the variation with respect to *K* was always approximately linear. Hence, we recommend that users take *K* = 100.

## Discussion

We do not claim that our package is an umbrella for other packages that analyze wrapped distributions. But other packages in R only implement the wrapped Cauchy, wrapped normal, wrapped skew normal, wrapped stable and wrapped normal mixtures distributions. Our package can compute the pdf, cdf, quantile function and random samples for any given parametric forms for *g* and *G* (that is, parametric forms for which the functions dg, pg, qg and rg are available in the base package of R or one of its contributed packages). Our package can also compute the following for 41 different wrapped distributions: maximum likelihood estimates of the parameters, standard errors, 95 percent confidence intervals, value of Cramer von Misses statistic, value of Anderson Darling statistic, value of Kolmogorov Smirnov test statistic and its *p*-value, value of Akaike Information Criterion, value of Consistent Akaike Information Criterion, value of Bayesian Information Criterion, value of Hannan Quinn Information Criterion, minimum value of the negative log likelihood function and convergence status when some data are fitted by the wrapped distribution. Hence, our package is a lot more applicable.

If the chosen *g* and *G* do not belong to one of the 41 distributions mentioned here, then our package will need updating to allow performing estimation. Nevertheless, the pdf, cdf, quantile function and random samples of the wrapped distribution can still be computed for the chosen *g* and *G* as long as the functions dg, pg, qg and rg are available in the base package of R or one of its contributed packages.

A future work is to develop similar R packages for bivariate and multivariate wrapped distributions. Another future work is to extend the package to cases when *g* is defined on domains different from the entire real line or when *g* is the probability mass function of a discrete random variable.
